# Reconstruction of the Swine Pulmonary Artery Using a Graft Engineered With Syngeneic Cardiac Pericytes

**DOI:** 10.3389/fbioe.2021.715717

**Published:** 2021-09-09

**Authors:** Valeria Vincenza Alvino, Anita C. Thomas, Mohamed T. Ghorbel, Filippo Rapetto, Srinivas A. Narayan, Michael Kilcooley, Dominga Iacobazzi, Michele Carrabba, Marco Fagnano, William Cathery, Elisa Avolio, Massimo Caputo, Paolo Madeddu

**Affiliations:** Bristol Medical School, Faculty of Health Sciences, University of Bristol, Bristol, United Kingdom

**Keywords:** pericytes, grafts, congenital heart disease, pulmonary artery, tissue engineering

## Abstract

The neonatal heart represents an attractive source of regenerative cells. Here, we report the results of a randomized, controlled, investigator-blinded preclinical study, which assessed the safety and effectiveness of a matrix graft cellularized with cardiac pericytes (CPs) in a piglet model of pulmonary artery (PA) reconstruction. Within each of five trios formed by 4-week-old female littermate piglets, one element (the donor) was sacrificed to provide a source of CPs, while the other two elements (the graft recipients) were allowed to reach the age of 10 weeks. During this time interval, culture-expanded donor CPs were seeded onto swine small intestinal submucosa (SIS) grafts, which were then shaped into conduits and conditioned in a flow bioreactor. Control unseeded SIS conduits were subjected to the same procedure. Then, recipient piglets were randomized to surgical reconstruction of the left PA (LPA) with unseeded or CP-seeded SIS conduits. Doppler echocardiography and cardiac magnetic resonance imaging (CMRI) were performed at baseline and 4-months post-implantation. Vascular explants were examined using histology and immunohistochemistry. All animals completed the scheduled follow-up. No group difference was observed in baseline imaging data. The final Doppler assessment showed that the LPA’s blood flow velocity was similar in the treatment groups. CMRI revealed a mismatch in the average growth of the grafted LPA and contralateral branch in both treatment groups. Histology of explanted arteries demonstrated that the CP-seeded grafts had a thicker luminal cell layer, more intraparietal arterioles, and a higher expression of endothelial nitric oxide synthase (eNOS) compared with unseeded grafts. Moreover, the LPA stump adjacent to the seeded graft contained more elastin and less collagen than the unseeded control. Syngeneic CP engineering did not accomplish the primary goal of supporting the graft’s growth but was able to improve secondary outcomes, such as the luminal cellularization and intraparietal vascularization of the graft, and elastic remodeling of the recipient artery. The beneficial properties of neonatal CPs may be considered in future bioengineering applications aiming to reproduce the cellular composition of native arteries.

## Introduction

Congenital defects of the pulmonary artery (PA) can occur as an isolated lesion or as an element of complex heart anomalies, as in the case of Tetralogy of Fallot. For patients with isolated valvular stenosis, balloon valvuloplasty is the treatment of choice; whereas patients with more complex defects require a palliative shunt intervention followed by, often multiple, attempts of definitive correction (Lenoir et al., 2017). Prosthetic or biological materials used for PA reconstruction are not ideal substitutes due to their thrombogenicity, limited durability, and inadequate growth and regeneration potential (Manavitehrani et al., 2019). Tissue engineering aims to create advanced therapy medicinal products (ATMPs) endowed with normal tissue function including capacity for growth and self-repair (Cheema et al., 2012; Durko et al., 2020; Majid et al., 2020). Cellularized vascular grafts can potentially overcome the shortcomings of currently used materials, and, if successfully passing all the development phases required for a marketing authorization, they might provide a better solution for the pediatric population. Several cell populations have been tested in animal models, including mesenchymal stromal cells, endothelial cells or induced pluripotent stem cells, yet the quest for the ideal cell type(s) is still open, as reviewed in (Avolio et al., 2015a; Jover et al., 2021).

The ideal cell product should confer the graft with rapid (re)endothelialization, which is critical for inhibiting thrombosis, and with remodeling capacity to match the characteristics of a normal artery and the rapid growth of the baby’s heart. Moreover, cell provision and manufacturing protocols should be tailored to the time of diagnosis and priorities in patient management. Cells from fetal tissues, such as the placenta, amnion, and umbilical cord, are best suited for cases diagnosed prenatally (Bongso et al., 2008; Pappa and Anagnou, 2009). In line with this, we reported the feasibility of using prosthetic conduits engineered with umbilical cord-derived mesenchymal stromal cells for the reconstruction of the PA in piglets (Ghorbel et al., 2019; Swim et al., 2019). In cases requiring a two-stage correction, cells from an accessible post-natal tissue, like the thymus (Iacobazzi et al., 2018; Albertario et al., 2019), or leftover material of palliative cardiac shunt surgery, could offer a scope to engineer available prostheses (Avolio et al., 2015b). Accordingly, we have proposed cardiac pericytes (CPs) as a cell source to engineer prosthetic grafts for the correction of right ventricle (RV) outflow defects (Alvino et al., 2020). In this pilot study, human or swine neonatal CPs were seeded onto decellularized swine small intestinal submucosa (SIS, CorMatrix) and cultured under static conditions for 5 days, followed by maturation of conduit-shaped SIS in a flow bioreactor. Immunohistological analyses showed the viability and integration of CPs in the outer layer of the conduit, and mechanical tests documented an improved elasticity of the CP-seeded SIS in comparison with the unseeded material (Alvino et al., 2020). Control and CP‐engineered conduits were then used to replace the left PA of piglets. After 4 months, anatomical and functional integration of the grafts was confirmed using Doppler echography, cardiac magnetic resonance imaging, and histology (Alvino et al., 2020).

To make a step forward in the translational pathway, we have now designed a randomized, controlled preclinical study aimed at testing the effectiveness of implanting CP-engineered conduits in piglets using the same surgical procedure employed to reconstruct the PA in humans. The endpoints were 1) the growth of the grafted PA, as assessed using cardiac magnetic resonance imaging (MRI) at baseline and 4-months follow-up, and 2) histological evidence of graft remodeling.

## Methods

### Data Communication Policy

The article adheres to the guidelines for implementation of the transparency and openness promotion of science. Following this policy, the authors will make the data, methods used in the analysis, and materials used to conduct the research available to any researcher for purposes of reproducing the results or replicating the procedure.

### Ethics

Institutional review board approval for the study was obtained according to the guidelines noted in the Journal Instructions to Authors. Animal experiments were performed in accord with institutional guidelines and followed the principles stated in the Guide for Care and Use of Laboratory Animals published by the National Institutes of Health in 1996 and the Animals (Scientific Procedures) Act published in 1986. The protocol was covered by the United Kingdom Home Office ethical approval PPL 30/3019 and PF6E6335D. The report of experimental data followed the Animal Research: Reporting of *In Vivo* Experiments (ARRIVE) guidelines (Percie du Sert et al., 2020).

### Manufacture and Quality Validation of Graft Cellularization

For each round of *in vivo* implantation, two SIS conduits (CorMatrix^®^ ECM^®^, CorMatrix Cardiovascular, Sunnyvale, CA) were prepared as described previously (Alvino et al., 2020), along with another conduit for quality validation of cell number and viability. In brief, CPs were isolated from swine hearts using an adaptation of the good manufacturing practice-compliant standard operating procedure previously used for human hearts. Single cell suspensions were sorted with anti-human immunomagnetic microbeads (Miltenyi Biotech, United Kingdom). Then, CD31^−^and CD34^+^ CPs were seeded and expanded to reach passage 4-5, in 4 weeks. The antigenic phenotype of expanded CPs was described previously (Alvino et al., 2020). These cells typically express the pericyte marker NG2, as well as the mural/mesenchymal markers platelet-derived growth factor receptor beta (PDGFRβ), α-SMA, vimentin, ecto-5-nucleotidase (eNT/CD73), and calponin (CALP), while being negative for CD146, CD31, and the hematopoietic marker CD45. Expanded cells were then used to cellularize an SIS sheet (seeding density of 20 x 10^3^ cells/cm^2^) while another sheet, cut from the same batch, was left unseeded. The optimal seeding density was validated previously, with the maintenance of cell viability being the decision criteria (Alvino et al., 2020). Both sheets were kept under static conditions for 5 days and then shaped into a conduit and conditioned in a flow bioreactor (3DCulturePro™ Bioreactors, TA Instruments, United Kingdom) at 24 ml/min for 7 days.

Conduit characterization was performed on samples from parallel conduits taken after bioreactor maturation and before surgery. The number of cells on the conduit was estimated using a CCK-8 assay (CCK8 Cell Counting Kit-8, Sigma). In brief, a standard curve was established using increasing concentrations of CPs in a 96-well plate (0–50,000 CP per well in duplicate). Two small pieces of each experimental conduit were placed into separate wells of the same plate immediately after removal from the bioreactor and covered with medium. A piece of bioreactor-matured unseeded conduit served as a negative control. After 2 h incubation with the CCK8 reagent the supernatant was removed from the wells containing the conduit pieces and the standard curve to measure the optical density (OD) using a Dynex Opsys MR microplate reader (Aspect Scientific, United Kingdom) set to 450 nm wavelength. The CPs from the standard curve wells were washed in PBS, trypsinized and counted. For calculation of the cell density, the area of conduits was measured using calipers and then the number of cells/cm^2^ was then calculated.

Cell viability was assessed using a CalceinAM/EthDIII live/dead assay (Biotium or Thermofisher, United Kingdom). Viable cells were observed using a Zeiss AxioObserver.Z1 microscope attached to a Leica DMi8 inverted epifluorescence microscope.

### *In Vivo* Protocol

**FIGURE 1:**
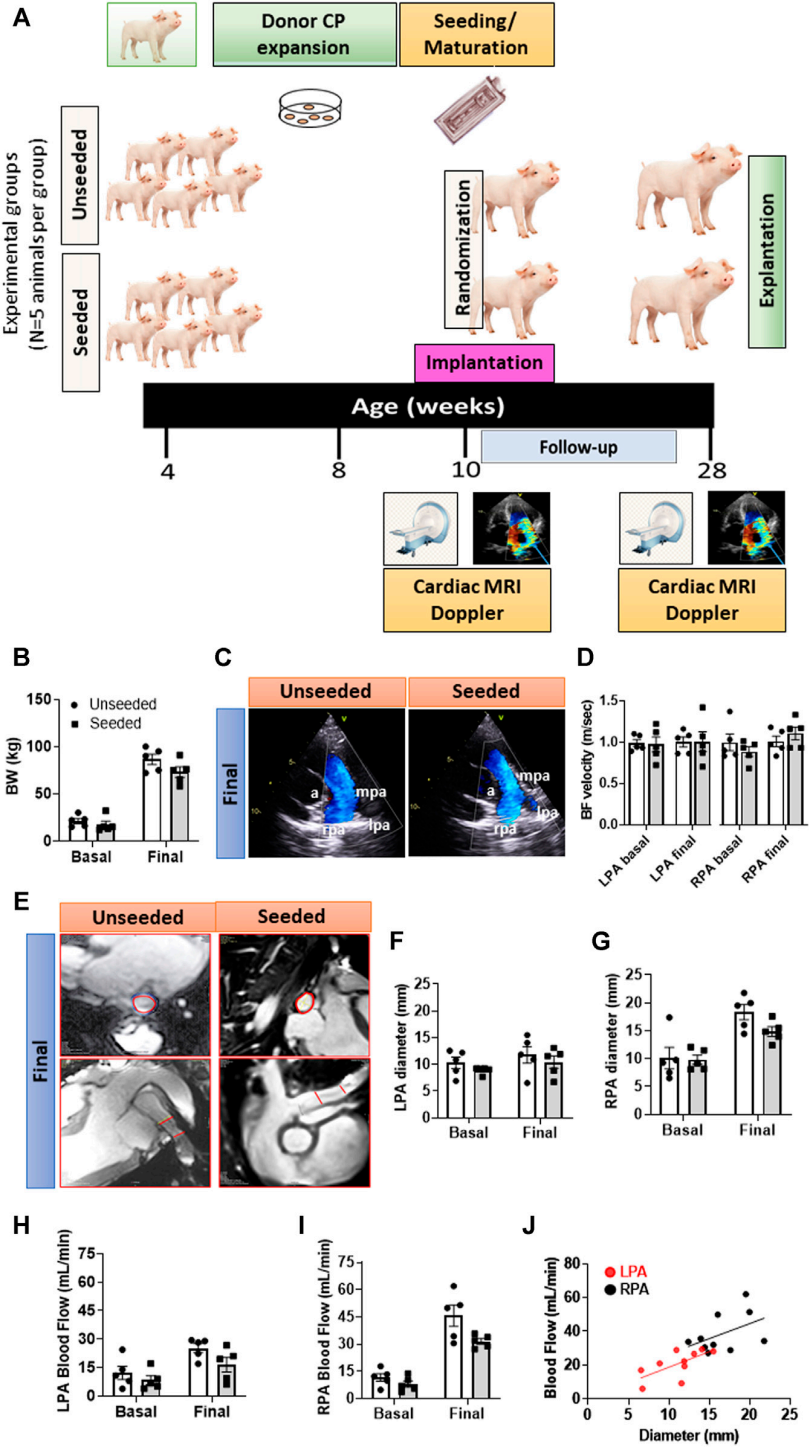
General outcome and imaging endpoints of the randomized, controlled preclinical study. **(A)** Schematic of the study. **(B)** Body weight of animals randomized to the two groups. **(C)** Representative images of Doppler echocardiography. Vascular structures are highlighted in the images: a = aorta, lpa = left pulmonary artery, mpa = main pulmonary artery, rpa = right pulmonary artery. **(D)** Column graph and individual Doppler data in the two groups at baseline and 4-moths follow-up. **(E)** Representative images of the cardiac MRI showing transversal and longitudinal sections of the site of grant implantation delimited by the red lines. **(F–I)** Column graph and individual MRI data (diameter and blood flow) in the two groups at baseline and 4-months follow-up regarding the LPA **(F,H)** and RPA **(G,I)**. **(J)** Correlation between diameter and blood flow regarding LPA and RPA. Abbreviations: BF = blood flow, BW = body weight. *N* = 5 biological replicates per experimental group. Data are expressed as mean ± SEM.

*Type of study:* A randomized, controlled, and operator-blinded pre-clinical study comparing swine implanted with CP-seeded or unseeded SIS conduits. Figure 1A illustrates a schematic of the study protocol.

*Endpoints:* Cardiac MRI indices of the PA dimensions and flow and histological evidence of graft remodeling.

*Randomization:* Within each of five trios formed by 4-week-old Landrace female littermate piglets, one element (the donor) was sacrificed to provide a source of CPs, while the other two elements (the graft recipients) were randomized to the two arms of the study, at 10 weeks of age, using a SAS Random Number Generator software. A total of 10 piglets (5 per arm) entered the study. One arm had the left PA (LPA) reconstructed with an unseeded SIS conduit and the other with a CP-cellularized SIS conduit (∼9 mm long and ∼6 mm in diameter). Each conduit had a code to allow the identification of treatment at completion of the data analysis.

*Surgery:* The procedure was conducted at the University of Bristol Translational Biomedical Research Centre (Langford) by a team composed of M.C. (principal surgeon) assisted by M.T.G. and F.R. All team participants were blinded to the group assignment. During surgery, the swine were maintained under general anesthesia and neuromuscular blockade. Animals recovered under intense postoperative monitoring for the initial 24 h. In brief, the pigs were observed continuously until awake and were then monitored closely at least every 2 h (chest drained, blood pressure, pulse, respiration, and temperature measured, and food, water, urine, and feces noted), with fluids IV continuously. Analgesia was given every 4 h IV and antibiotics every 6 h. When not observed directly, the animals remained under observation using close-circuit TV.

*Follow-up:* Imaging studies were performed at baseline and 4 months after implantation using a cardiac MRI 3‐T scanner (Siemens Healthcare, Erlangen, Germany) and data were analyzed by an investigator blinded to the randomization protocol (S.N.). The heart and pulmonary arteries were also examined using 2‐dimensional Doppler echocardiography (VividQ; GE Healthcare, Cardiff, United Kingdom), and flow velocity within the graft and nearby vessels measured by an investigator blinded to the randomization protocol (M.G.). At sacrifice, the LPA and other tissues were harvested and processed. Sections were used for histology, immunohistochemistry, and morphometric analyses, as described in detail previously (Alvino et al., 2020).

### Immunohistochemistry of Explanted Swine Grafts

Immunohistochemical analyses of LPA stumps and adjacent grafts were performed separately by circumscribing the two tissue areas according to the suture point and the difference in the immunohistochemical coloring of the intima and media layers of the native LPA.

*Tissue embedding and processing:* The conduits harvested from the animals were opened out, cut into small pieces, and fixed in 4% (w/v) paraformaldehyde (PFA). Samples were put in 30% (w/v) sucrose, embedded in optimal cutting temperature (OCT) compound to obtain frozen tissues, or processed using the Excelsior AS automated processor (Thermo Fisher, United Kingdom) and embedded in paraffin. Negative controls for immunohistochemistry included tissue sections without primary antibodies as technical internal control. Positive control samples that are not indicated in this manuscript were shown previously (Alvino et al., 2020). Analyses were performed using the whole section area, on at least two non-consecutive sections per animal.

*General overview of the tissue’s structure:* Hematoxylin and eosin (H&E) and Verhoeff’s elastic stain (EVG) stainings were performed on frozen sections to assess the tissue’s structure and to measure the luminal side of the grafts, the distribution of cells, and the composition of the extracellular matrix (ECM). Frozen 8-μm-thick sections were cut using the cryostat STAR Nx50 (Thermo Fisher, United Kingdom) set at −20°C and fixed in ice-cold glacial acetone (Fisher, United Kingdom) for 5 min at −20°C. Fixed sections were kept air-dried for 15 min and hydrated in 1xPBS for 10 min at RT. Staining was performed using a Shandon Varistan 24–4 slide stainer (Thermo Fisher, United Kingdom), according to the manufacturer’s instructions. Slides were mounted with DPX, covered with coverslips, and air-dried. Images were taken using a Zeiss Observer. Z1 microscope set up on a bright field light path. Tiling parameters of the Zen Pro software were used to cover the whole LPA stump and graft using 2.5x and 10x objectives, while inserts were acquired at higher magnification, as indicated in illustrative figures. The area of EVG (purple-black) staining was expressed as a percentage of the total area using a morphometric analysis with Image J software (http://rsbweb.nih.gov/ij/).

*Assessment of collagen:* Five-μm-thick paraffin-embedded sections were cut using a Shandon Finesse 325 manual microtome (Thermo Fisher, United Kingdom) and assessed for fibrosis using the Azan Mallory histochemical technique. Before using immunohistochemistry, the paraffin-embedded sections were subjected to the antigen retrieval to unmask the epitopes within the tissue, using citrate buffer 0.02 mol/L, pH = 6 for 30 min at +98°C. All the images, inserts, and tiling parameters were taken using the same microscope indicated above. Collagen deposits in the form of blue staining within the LPA stump and adjacent graft were quantified as the percentage of the total area using Image J software for the morphometric analysis. The ratio of elastic fibers to collagen was then calculated.

*Quantification of intraparietal vascular smooth muscle cells:* Paraffin-embedded sections were used to assess the newly formed muscular layer underneath the luminal endothelium of the graft. For the detection of vascular smooth muscle cells (VSMCs), anti-α-SMA-cy3 conjugated (1:400, Sigma-Aldrich, United Kingdom) was incubated with tissue sections for 16 h, at +4°C. Nuclei were stained with (v/v) DAPI solution and then sections were mounted with Fluoromount G. Fluorescent images were taken using the Zeiss Observer. Z1 microscope with 10x and 20x objectives set up on the fluorescent light path as indicated in the figures.

*Assessment of the endothelialization and quantification of microvascular density:* Endothelial cells (ECs) covering the LPA intima and the adjacent graft were recognized using anti-human CD31 (1:50, R&D systems, United Kingdom) for 16 h at +4°C. We have previously demonstrated that anti-human CD31 selectively binds this transmembrane receptor expressed on porcine endothelial cells. In addition, the use of a highly adsorbed secondary antibody conjugated to CD31 ensures a high level of IHC specificity in the lumen of endothelial cells (Alvino et al., 2020). Frozen sections were incubated with Alexa Fluor 647-conjugated highly adsorbed anti-mouse IgG (1:200, for 1 h, at +20°C, Life Technologies, United Kingdom). Slides were stained with 1:1,000 (v/v) DAPI solution in 1xPBS and mounted with Fluoromount G for the imaging. Representative images were taken with 20x objectives using Zeiss Observer. Z1 fluorescent microscope*.* In addition, paraffin-embedded sections were incubated with anti‐isolectin GS‐IB4, which detects the sugar residues of the EC glycocalyx (Life Technologies, United Kingdom, 1:200), followed by Alexa Fluor 488 Streptavidin secondary antibody (Invitrogen, United Kingdom, 1:200, 1 h, RT). For the assessment of intraparietal microvessels, the sections were stained with anti-human CD31 and anti‐isolectin GS‐IB4 to recognize ECs. In arterioles, VSMCs were identified by staining with anti-α-SMA cy3-conjugated antibody, as for the quantification of intraparietal VSMCs. Nuclei were stained with (v/v) DAPI solution and then sections were mounted with Fluoromount G. The density of capillaries and arterioles (number per mm^2^) was assessed for the whole section area comprising the LPA stump, from where the vasculature originates, and the adjacent graft. The vascular densities of the two groups were then compared separately within the stump and the graft.

*Quantification of NG2+ vessels:* For the detection of pericytes, frozen sections were incubated with an anti-swine NG2 (1:50, NovusBio, United Kingdom) followed by Alexa Fluor 488-conjugated anti-rabbit IgG secondary antibody. The same sections were treated with anti-human CD31 and anti-α-SMA, all incubated for 16 h at +4°C, to discriminate the localization of mural cells either in the media, adventitia, or in the basement membrane. Vessels covered with NG2+ pericytes were expressed as a percentage of total capillaries and arterioles.

*Quantification of eNOS expression:* Paraffin-embedded sections were stained with anti-human endothelial nitric oxide synthase (eNOS) primary antibody (1:100, Abcam, United Kingdom). The presence of the molecule in the form of brown precipitates was revealed using the REAL EnVision Detection System, 3,3′-Diaminobenzidine (DAB) kit (Dako, United Kingdom), according to the manufacturer’s instructions. The images were visualized using the light microscopy of the Olympus BX40 and QCapture Pro 5.0 software. For the quantitative analysis of eNOS, we used ImageJ software (http://rsbweb.nih.gov/ij/), converted the RGB images to an 8-bit grayscale image, and then used the threshold tool to set the threshold color (red channel) corresponding to the area of dark brown eNOS in the vessels and the endothelial layer of the graft and LPA Stump. We used the Measure Tool to calculate the area, which is the size of the selection within the set threshold. To calculate the percentage of eNOS area, we divided the dark brown precipitate area by the total area of the picture. Data were expressed as the percentage of the total area in mm^2^. Cross-sections of human placental villi were used as a positive control to detect eNOS in syncytiotrophoblasts.

### Statistical Analysis

The data were collected according to the intention-to-treat concept. (Gupta, 2011; McCoy, 2017). Statistical significance for differences between the two experimental groups was determined using Student’s *t*-test. When assessing the effect of two variables and their interaction, two-way ANOVA, with post-hoc Sidak’s test was used. Data are expressed as individual values and means ± SEM. Probability values (*P*) < 0.05 were considered significant.

## Results

### Quality Validation of Graft Cellularization After Bioreactor Conditioning

The viability of CPs seeded in the SIS conduit was 96 ± 2% after 5 days of static culture and 91 ± 2% after 7 days of conditioning in the bioreactor (Supplementary Figure S1). The cell density for each CP-seeded conduit immediately before engraftment was 84 ± 14 x 10^3^ cell/cm^2^. Both seeded and unseeded conduits were formed into custom-sized grafts (9.2 ± 0.3 mm long and 7.2 ± 0.2 mm diameter) immediately before implantation.

### General Outcome and Imaging Endpoints of the Randomized, Controlled, and Blinded Preclinical Study

All the animals concluded the 4-months follow-up, with a similar body weight increase in the two arms of the study (Figure 1B).

There was no difference in the pre-implantation imaging data between the two groups, thus excluding any imbalance in baseline characteristics causing chance bias. Analysis of data from the final Doppler did not reveal any group difference in the blood flow velocity regarding the grafted LPA or untouched contralateral right branch (RPA) (Figures 1C,D). Representative images of the cardiac MRI are shown in Figure 1E. The LPA anteroposterior diameter remained unchanged from baseline to the final assessment in both groups, whereas the untouched RPA grew over the same period by 1.79-fold in the unseeded (*p* < 0.01) and by 1.52-fold in the seeded group (*p* < 0.05) (Figures 1F,G). Blood flow increased during the follow-up, although more remarkably in the untouched RPA (*p* < 0.001) than in the grafted LPA (*p* < 0.05), with no difference between seeded or unseeded groups (Figures 1H,I). The slope of the relationship between flow and diameter was similar in the LPA and RPA, although individual points of the LPA were shifted to the lower-left corner for the RPA (Figure 1J). These data indicate that the grafted conduit had limited growth potential, which resulted in a reduction of the corresponding blood flow. Moreover, these limitations could not be avoided by the presence of CPs in the conduit.

### Immunohistochemistry Endpoints

Although the imaging methods could not demonstrate the efficacy of the engineered conduit, histological studies revealed several important differences between the two arms of the study. We compared the cellular composition, intraparietal vascularization, and ECM remodeling of seeded and unseeded grafts and attached LPA.

**FIGURE 2:**
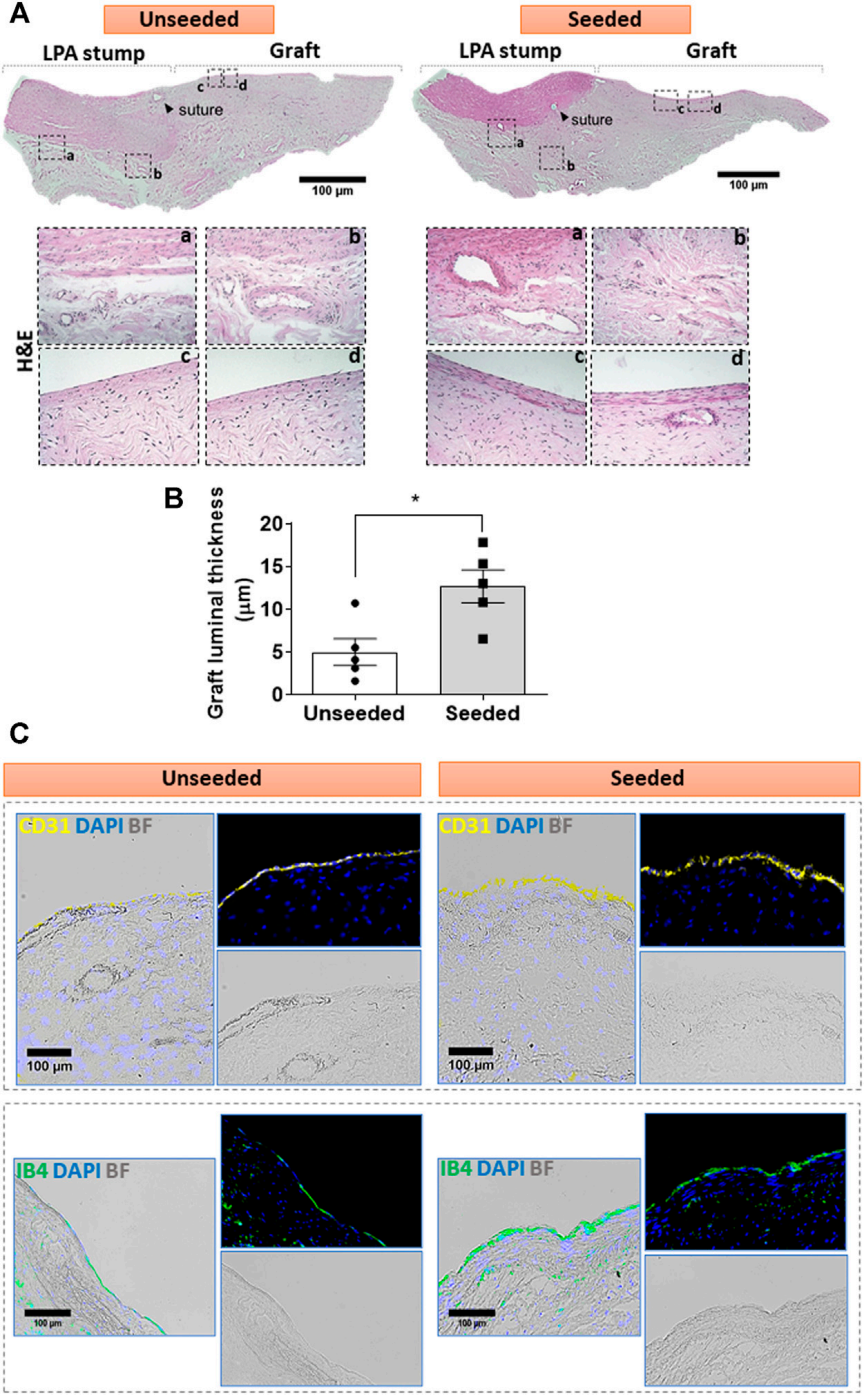
Cellular composition of the graft: **(A)** H&E staining of unseeded and seeded grafts. Tiled images and inserts show the general architecture and details of explanted grafts, including the distribution of cells (cell nuclei are dark blue and eosinophilic compounds in the cytoplasm are pink) and the ECM underneath the luminal cell layer, containing fibroblasts and microvessels. **(B)** Bar graph showing the thickness of the luminal layer. *N* = 5 biological replicates per experimental group. Data are represented as mean ± SEM. *p < 0.05. **(C)** Immunofluorescent and bright-field images display the presence of CD31 (yellow) and IB4 (green) markers in the luminal endothelium of the grafts. Representative images are acquired using 2.5x, 10x, and 20x objectives.

**FIGURE 3:**
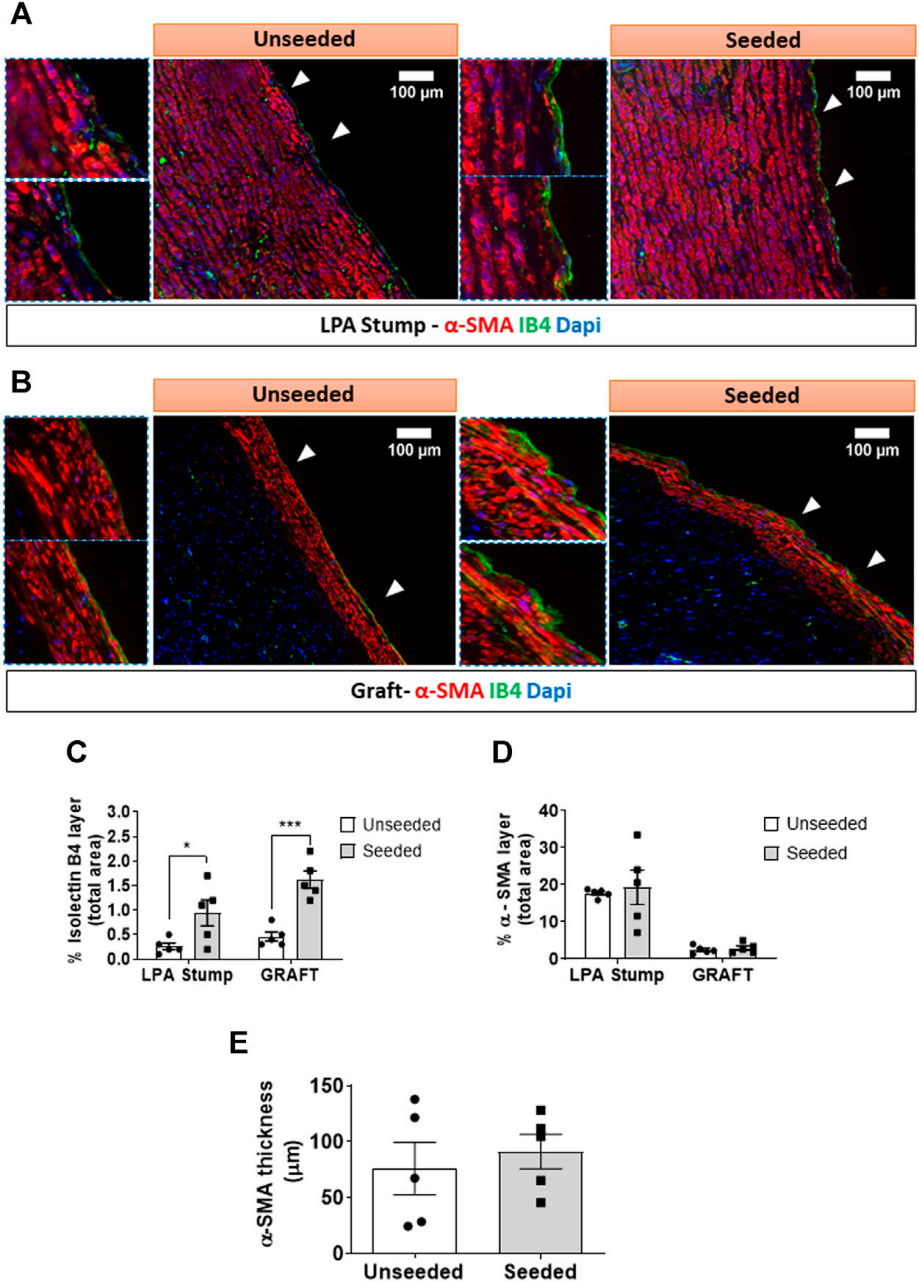
Multicolor fluorescent microscopy shows the luminal layer of the graft is composed of endothelial and smooth muscle cells. **(A,B)** Images showing the layer of luminal endothelial cells (green) sitting on several sheets of α-SMA + VSMCs (red) at the level of the LPA stump **(A)** and the grafts **(B)**. Bar graphs showing the quantification of IB4 **(C)** and α-SMA staining measured as percentage of positive expression in the internal layer of total section area **(D)**. α-SMA was also calculated as thickness layer expressed in μm **(E)**. The images were visualized using a fluorescent microscope with 10x objectives and bigger inserts were added for better visualization of the details. *N* = 5 biological replicates per experimental group. Data are expressed as mean ± SEM. *p < 0.05, ***p < 0.0005.

*Cellular composition of the graft:* The H&E staining demonstrated the structural continuity between the graft and the attached LPA stump, with a demarcation indicated by the presence of suture points. As shown in Figure 2A, the LPA stump was composed of three distinct layers, the intima, tunica media, and abluminal adventitia. The graft showed a luminal layer of longitudinally oriented cells resting on connective tissue containing microvascular structures and fibroblastic cells oriented along the ECM fibers. The morphometric analysis of the luminal layer denoted this was 2.5-fold thicker in seeded grafts as compared with unseeded grafts (Figure 2B). We next used immunofluorescent microscopy to characterize the cell populations at the level of the lumen and underneath layer. Staining for CD31, which labels a glycoprotein expressed on EC, and IB4, which binds to the sugar residues of the endothelial glycocalyx, demonstrated stronger signals, as assessed using Image J, in the seeded grafts compared with the unseeded grafts (Figure 2C). Moreover, using a combination of fluorescent markers, we identified VSMCs were located underneath the IB4 positive endothelium, in continuity with the tunica media of the LPA stump (Figures 3A–E). Cellularized grafts showed a significant increase of the IB4 staining in the luminal side (Figure 3C), whereas the fluorescence intensity and thickness of the α-SMA positive layer were similar between the two groups (Figures 3D,E).

**FIGURE 4:**
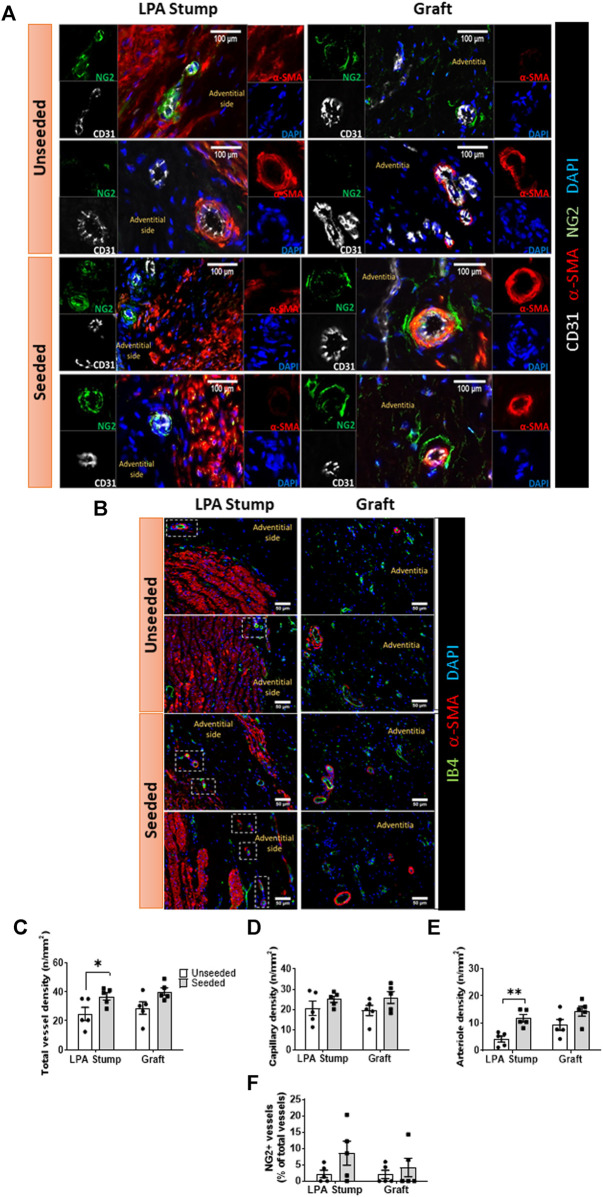
Microvascular composition of the implanted artery. **(A,B)** Immunofluorescent microscopy image of the vascularization at the level of the LPA stump and grafts. In panel A, endothelial cells were stained with CD31 (white), vascular smooth muscle cells with a-SMA (red), pericytes with NG2 (green), and nuclei with DAPI (blue). In panel B, endothelial cells stained with IB4 (green), vascular smooth muscle cells with α-SMA (red), and nuclei with DAPI (blue). The location of the neo-adventitia is indicated in each image. Vascular smooth muscle cells are visualized in the tunica media of the LPA stump, the graft structure, and within intraparietal arterioles. **(C–F)** Bar graphs showing the vascular density and pericyte coverage in the two groups at the level of the LPA stump and grafts **(C)** Total vascular density, **(D)** Capillary density, **(E)** arteriole density, **(F)** NG2+ percentage. *N* = 5 biological replicates per experimental group. Data are expressed as mean ± SEM. *p < 0.05, **p < 0.01.

*Vascular density:* The animals implanted with cellularized grafts had a higher vascular density in the total explant area compared with the unseeded controls (*p* < 0.01) (Figures 4A,B). The analysis of distinct areas of the explant revealed that the increase in vascular density occurred at both the LPA stump (*p* < 0.05) and the graft (*p* = 0.05) level (Figure 4C). This could be attributed to the heightened arteriole density (*p* < 0.01), whereas capillary density was similar between seeded and unseeded grafts (Figures 4D,E). Several vessels within the LPA stump and the graft were surrounded by NG2+ pericytes. However, the fraction of NG2+ vessels was similar in the two treatment groups (Figure 4F).

**FIGURE 5:**
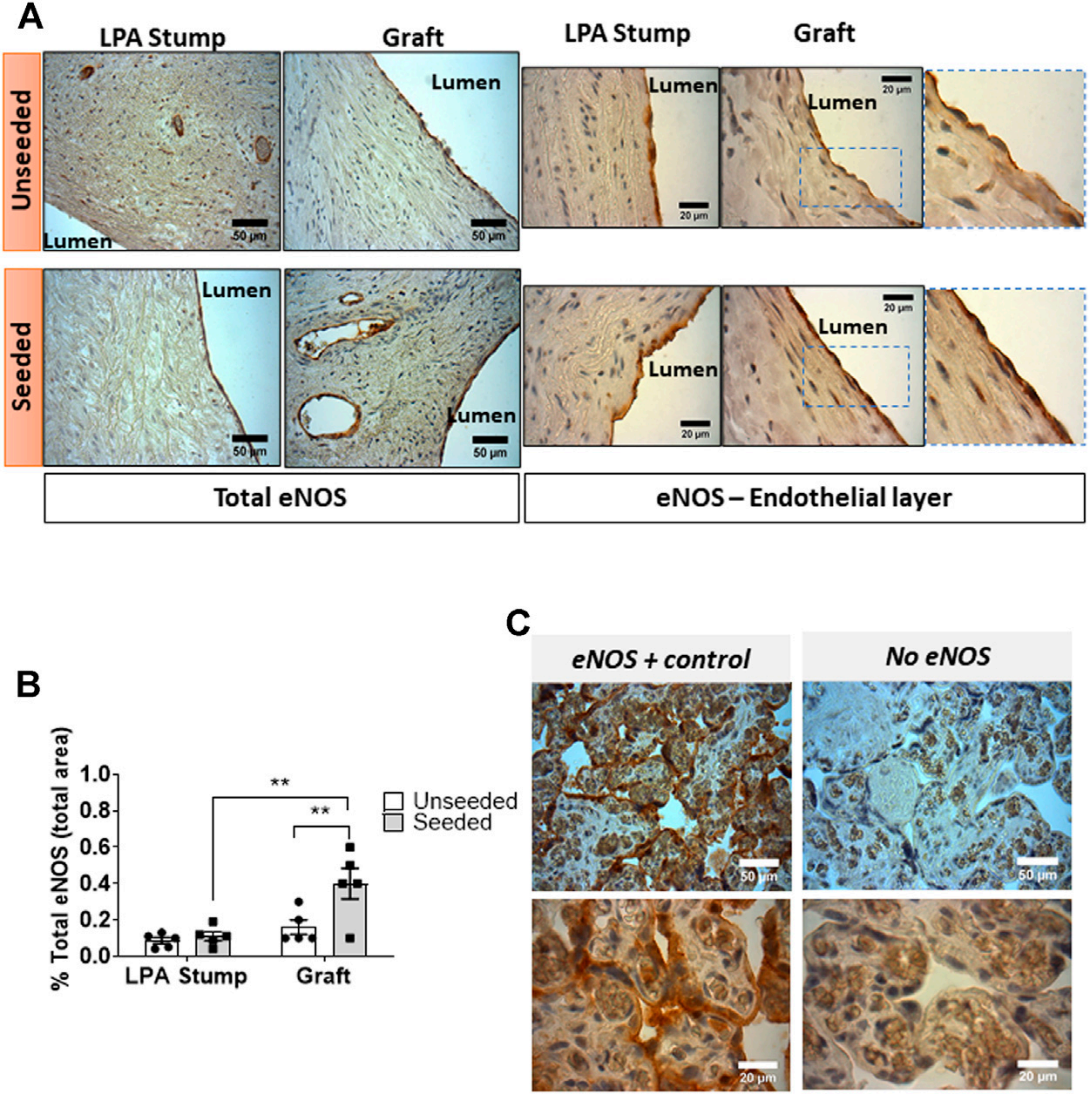
Expression of eNOS expression in the implanted artery. **(A)** Light microscopy images displaying the expression of eNOS in the LPA stump and graft in the form of brown precipitates. An intense staining, continuous staining was detected in endothelial cells of the seeded grafts (both at the luminal side and in intraparietal microvessels) compared with unseeded grafts. Moreover, weak positive staining could be appreciated in muscular cells underneath the luminal endothelial cells of the seeded grafts. **(B)** Bar graph showing the quantification of eNOS staining measured as the percentage of total section area. Images were quantified using a light microscope with 40x and 100x objectives. *N* = 5 biological replicates per experimental group. Data are expressed as mean ± SEM. **p < 0.01 **(C)** Cross-sections of human placental villi (used as a positive control) show the strong expression of eNOS in the syncytiotrophoblasts.

*Expression of eNOS:* Immunohistochemistry analysis showed the expression of eNOS by ECs of the luminal layer and intraparietal microvessels of the LPA stump and graft (Figures 5A,B). Sections of placenta villi were used as a positive control (Figure 5C). Using two-way ANOVA, we detected a difference in the global eNOS expression between the two groups, with the posthoc test confirming a higher expression in seeded grafts compared with unseeded controls (*p* < 0.01). Interestingly, the explanted seeded grafts showed 2.5-fold higher levels of eNOS compared with corresponding stumps (*p* < 0.01).

Moreover, two major differences were observed when zooming into the luminal layer of the grafts (magnification inserts of Figure 5A). First, the eNOS staining was continuous in ECs of the seeded graft, at variance with the sporadic characteristic observed in the unseeded graft. Second, VSMCs underneath the ECs of seeded grafts were weakly positive for eNOS, whereas no signal was detected in the unseeded controls.

**FIGURE 6:**
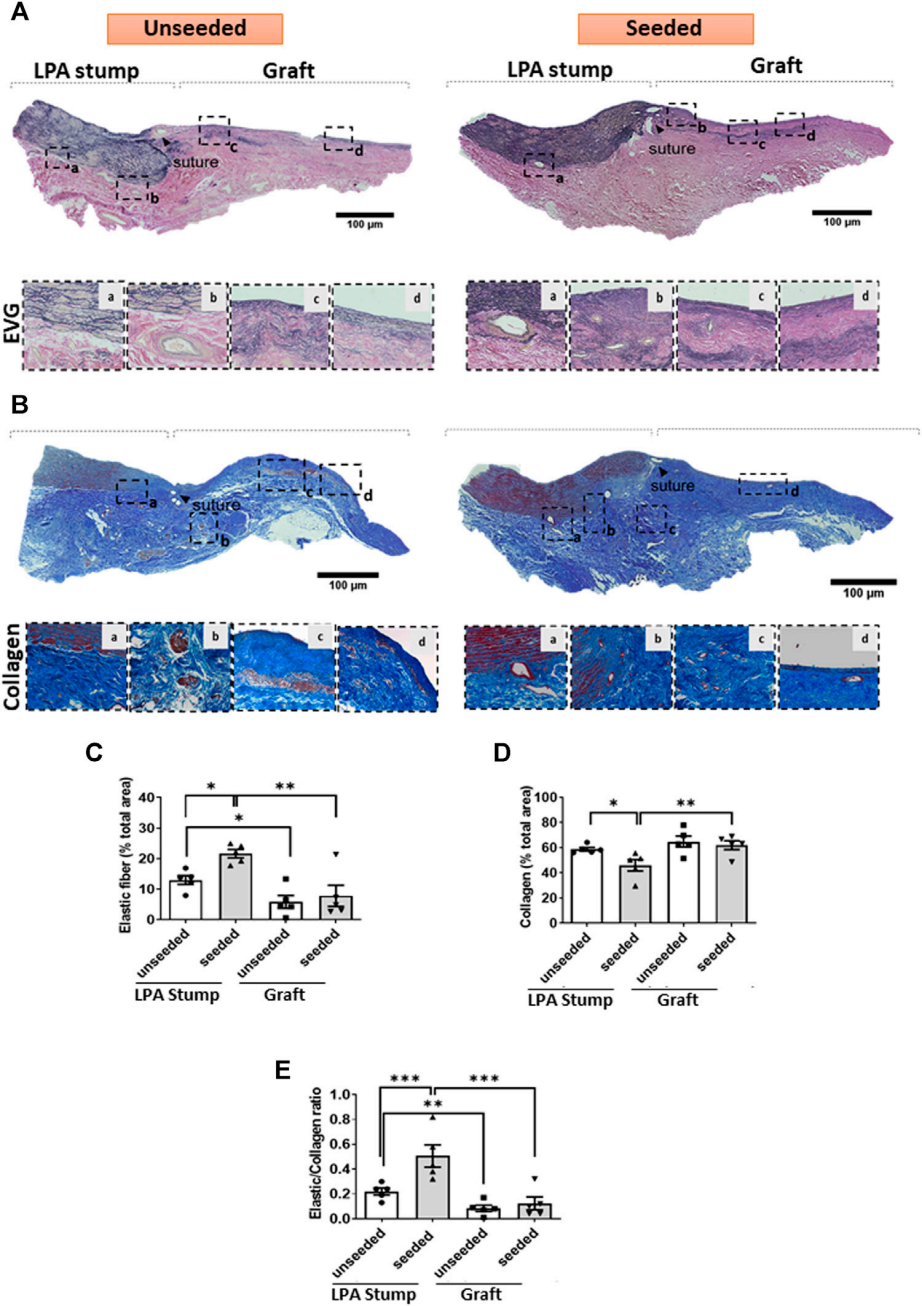
ECM remodeling of the implanted artery. **(A,B)** Tiled images and inserts at higher magnification display the composition of elastic fibers of the LPA and graft, as assessed using EVG **(A)** and Azan Mallory **(B)** stainings. **(C–E)** Bar graphs showing the results of the quantification of elastin and collagen and their ratio. Images were visualized using a light microscope with 2.5x, 10x and 20x objectives. *N* = 5 biological replicates per experimental group. per experimental group. Data are expressed as mean ± SEM. *p < 0.05, **p < 0.01, and ***p < 0.001.

*Extracellular matrix remodeling:* The LPA stump adjacent to the seeded graft had a higher content of elastic fibers, as denoted by the EVG staining, and less collagen, identified by Azan Mallory staining, compared with controls (Figures 6A–E), with these reciprocal changes resulting in a remarkable increase in the elastic to collagen content ratio in the LPA stump (Figure 6E). As expected, the implanted grafts had lesser elastic fiber content than the corresponding LPAs, with no difference between seeded and unseeded grafts (Figures 6C,E).

## Discussion

The present study is the first to be conducted using a graft cellularized with syngeneic CPs to reconstruct a branch of the piglet PA. The model is intended to reproduce the surgical method used for the correction of congenital anomalies of the PA. Moreover, we attempted to simulate the clinical scenario where CPs are isolated from the young heart, expanded, and implanted within a graft into the PA. In this set of experiments, we obtained swine donor cells from a recipient’s sibling to avoid the stress caused by a double intervention.

### Feasibility and Safety

Results of the preclinical study confirm that the tissue engineering protocol is feasible. Moreover, the safety of implantation was demonstrated by the normal growth of the swine, the absence of thrombotic events, and the persistent patency of the grafted PAs.

### Primary Endpoints of Efficacy

The assessment of the primary endpoints at the 4-months follow-up did not provide evidence of efficacy. In both arms of the study, the cardiac MRI showed that the grafted LPA did not grow to the same extent of the untouched RPA, with the lack of growth resulting in a defective blood flow through the grafted artery. No significant difference was observed between the two treatment groups regarding both the pre-implantation and final MRI and Doppler data. The similarity in baseline data excludes the possibility that a chance bias in the initial randomization has compromised the robustness of the efficacy analysis.

In our previous study, we anticipated that sample size should be set at 14 (7 swine per group) (Alvino et al., 2020). A study of this size would have 80% power to detect a difference in the Doppler blood flow velocity of 0.3 ml/min with an SD of 0.2.11 Considering the ethical and financial challenges associated with the use of swine, we decided to perform an ad interim recalculation of the group size and cost/benefit ratio after completion of 10 animals (5 per group) before engaging in a larger study. The recalculation of the SD for the Doppler blood flow velocity confirmed a value (0.23) close to the initial assumption (0.20) but with a null effect size (0.002). The SD for the MRI assessment of the antero-posterior diameter of the LPA was 3.01, with an effect size of 1.35 favoring the unseeded graft group compared with the seeded graft group. Therefore, we concluded there was no justification to extend the study.

### Secondary Endpoint: Improvement of Endothelialization and Intraparietal Vascularization

Immunohistochemistry showed that bioengineering of the SIS graft with swine CPs resulted in a thicker intimal layer and a more abundant intraparietal vascularization. These results are compatible with the *in vitro* data of our previous study showing swine CPs can recruit ECs and promote endothelial network formation through the secretion of paracrine factors (Alvino et al., 2020).

Graft endothelialization is crucial to maintain an anti-thrombotic surface and preserve the vasodilatory ability of the reconstructed artery, while vascularization is necessary for the maintenance of perfusion within the vessel wall (Chang and Niklason, 2017). Methods to achieve these goals consist of seeding autologous ECs onto vascular prostheses or decorating them with angiogenic/chemoattractant factors to recruit ECs from the anastomotic sites and proangiogenic monocytes from the circulation (De Visscher et al., 2012; Lee et al., 2013; Smith et al., 2020). However, these methodologies involved additional manufacturing steps and showed incomplete endothelialization especially in the center of the grafts (Smith et al., 2020). Therefore, the ability of CP-engineered grafts to promote spontaneous enhancement of the luminal cellularization and intraparietal vascularization represents a success of the present preclinical study.

Regarding the underpinning mechanisms, two possibilities should be considered 1) the seeded cells and/or their extracellular matrix were entirely responsible for the recruitment of ECs or 2) the seeded cells have synergized with the CorMatrix’s native angiogenic properties. In support of the second possibility, the epicardial implantation of non-cellularized CorMatrix in a porcine model of coronary ischemia-reperfusion attenuated the maladaptive remodelling following MI and promoted healing and functional recovery; in addition, the matrix reportedly promoted vasculogenesis in the region of functional recovery (Mewhort et al., 2016). Another study reporting the use of non-cellularized SIS-ECM was carried out in a rodent MI model: implantation of an active SIS-ECM patch on the epicardium had a therapeutic effect on the cardiac remodelling and functional recovery compared with SIS-ECM that had been inactivated by exposure to glutaraldehyde or guanidine hydrochloride (Mewhort et al., 2017). The SIS-ECM induced a strong vasculogenic response through the formation of functional blood vessels when in contact with endogenous fibroblasts, with FGF-2 being the key regulator of the bioactive effects. This cumulative evidence suggests that acellular SIS-ECM has intrinsic regenerative capacities.

We attempted to assess the contribution of seeded cells by verifying their persistence in two grafts that were explanted 12 or 14 days after implantation. In this pilot study, two female swine were used as recipients and CPs were derived from a brother. Fluorescent *in situ* hybridization (FISH) was used to identify cells expressing the Y chromosome using a protocol described by us previously (Albertario et al., 2019), with the exception of the probe, which was purchased from another vendor (Chromosome Science Labo, Japan) because the one used in (Albertario et al., 2019) had been discontinued. Unfortunately, the study was unsuccessful because the Y chromosome could not be detected in a positive control (male heart biopsy). This represents a limitation of our study precluding an interpretation of the seeded cells’ persistence after implantation *in vivo*.

Re-endothelialization was associated with an increased expression of eNOS. The eNOS staining was strong in ECs at the luminal side of the graft and the endothelium of intraparietal vessels but could also be appreciated in VSMCs underneath luminal ECs. The enzyme eNOS is a source of NO, which diffuses to the VSMCs to induce vasorelaxation (Gao et al., 2016). Recent studies have suggested that the VSMCs express eNOS and that VSMC-derived NO can account for vasodilation even in endothelium-denuded conditions (Han et al., 2013). Since the pulmonary circulation is operating with distinctively low arterial pressure, the synergistic effect of EC- and VSMC-derived eNOS might be underlying the characteristic low PA resistances (Kim et al., 2019). Reduced expression of eNOS has been associated with alterations in the structure and endothelial function of the pulmonary vasculature in smokers and children with congenital heart defects (Barberà et al., 2001; Ferreiro et al., 2001). To the best of our knowledge, this is the first report indicating that bioengineering with CPs can induce long-term upregulation of eNOS expression in the vascular graft. Further studies are necessary to determine the mechanism underpinning eNOS induction and the clinical implication of this phenomenon.

### Secondary Endpoint: Elastic Remodelling

Another significant result of graft bioengineering consisted of the elastic remodeling of the LPA stump. Pulmonary vascular stiffness refers to an elastic PA’s innate resistance to deformation under a pressure load and it mainly depends on the vessel’s inherent material properties. Elevations in stiffness due to the reduction of the elastic content have been associated with increased adult mortality and poorer pediatric outcomes (Mahapatra et al., 2006; Hunter et al., 2008). This association is believed to be due to several factors, including flow inefficiency, increased right ventricular afterload independent of pulmonary vascular resistance, and exacerbation of vascular pathologies (Hunter et al., 1985).

Results of mechanical tests from our previous study suggested that CorMatrix conduits acquired a more elastic behavior when seeded with sCPs, as denoted by a significant decrease in Young’s modulus, but also an increased strain at rupture (Alvino et al., 2020). However, these data could not predict the primary outcomes of the present study, thus suggesting that mechanical tests require further refinement, including the comparison of pre- and post-implantation features. This double assessment was not done in the present study and represents therefore a limitation.

### Critical Comparison of Results With Other Tissue Engineering Strategies

The failure in achieving the primary endpoint despite positive secondary outcomes represents an intriguing aspect of the study. While endothelialization and intraparietal angiogenesis are important regenerative features, other mechanisms are necessary for the eutrophic remodelling of the implanted graft. In recent years, several bioengineering approaches have been proposed to reach this goal.

We have previously shown that fetal tissues can be a valuable source of stromal cells for tissue engineering (Avolio et al., 2015a). The advantage of CPs over umbilical cord blood-derived stromal cells consists of their superior purity and defined antigenic phenotype. Moreover, the differentiation of stromal cells into VSMCs involves an additional step in manufacturing, which results in increased complexity and delayed availability, both detrimental for cases requiring rapid correction. On the other hand, VSMCs derived from the differentiation of mesenchymal stromal cells conferred conduits with the growing capacity that was precluded to CPs (Ghorbel et al., 2019; Iacobazzi et al., 2021). Considering that human CPs can be also induced to contractile VSMCs during culture expansion (Ghorbel et al., 2019), it would be interesting to determine if seeding SIS grafts with CP-derived VSMCs may improve the graft capacity to remodel and grow after implantation *in vivo*.

Functional VSMCs could be efficiently generated on a large scale from induced pluripotent stem cells (iPSCs). Following implantation into a rat aortic model, hiPSC-engineered vascular grafts showed excellent patency without luminal dilation and effectively maintained mechanical and contractile function (Luo et al., 2020). Apart from mural cells, grafts cellularized with autologous ECs (Ma et al., 2017) and endothelial progenitor cells (Yuan et al., 2020) were used in preclinical models. This approach is intended to reduce the risk of early thrombosis and confer the graft with immediate remodelling capacity (Carrabba and Madeddu, 2018).

Alternative methods using cells as a source of biocompatible matrix and novel bioprinting devices have been developed in recent years. For instance, Syedain et al. (2016) showed that off-the-shelf tubes of fibroblast-derived collagenous matrix can regenerate and grow as a PA replacement in synchrony with body growth in lambs. Recently, they implemented the product to generate a valved conduit consisting of three tubes sewn together (Syedain et al., 2021). The tri-tube valves demonstrated resistance to calcification and improved hemodynamic function compared to clinically used pediatric bioprosthetic valves tested in the same model (Syedain et al., 2021). Similarly, acellular grafts made of synthetic material exploit the patient’s body as a living bioreactor for cellularization *in vivo*. However, the endogenous cells’ colonization process is slow, making these synthetic grafts prone to thrombosis and deformation (Pashneh-Tala et al., 2016).

Grafts made of decellularized natural blood vessels are superior regarding biocompatibility. However, the process of decellularization can damage the extracellular matrix. To circumvent this limitation, synthetic material has been used to create a reinforced hybrid vascular graft that was used in a rat aorta interposition model (Gong et al., 2016; Ran et al., 2019). New automated technology based on dip-spinning methodologies were proposed to manufacture constructs with native mechanical properties and cell-derived biological activities, critical for clinical graft applications (Akentjew et al., 2019).

## Conclusion

Bioengineering of SIS grafts with syngeneic CPs resulted in some improvements regarding endothelialization but failed in the goal of conferring the graft with growth capacity. Cell-engineered grafts hold promises, but none has reached full clinical application. The main reason being they failed to fully reproduce the structure/function of native arteries. Accordingly, we are already working on a second-generation graft, made of a three-layered structure decorated with specialized vascular cells, including EC, VSMCs, and adventitial pericytes, aiming to mimic the characteristics of the PA.

## Implications of the Study

1) What is new?
  • We have provided evidence for the feasibility of using a pericyte-cellularized SIS conduit for the reconstruction of the pulmonary artery in piglets.
  • Pericyte engineering improves the *in vivo* luminal cellularization and intraparietal vascularization of SIS grafts, potentially through long-term induction of endothelial nitric oxide synthase. In addition, the engineered product confers the recipient artery with elastic features. However, the study showed no benefit the vessel growth.
2) *What are the clinical implications?*
  • Cardiac pericytes available from leftover tissue of palliative surgery could be used to engineer clinical-grade matrix conduits ready for implantation at the occasion of definitive correction of the pulmonary artery defect. This solution can accelerate the colonization of the implanted graft by the recipient’s cells to acquire characteristics of the native pulmonary artery.

## Data Availability

The original contributions presented in the study are included in the article/Supplementary Material, further inquiries can be directed to the corresponding authors.

## Supplementary Material

Supplementary Figure S1 Cell viability of CorMatrix grafts engineered with swine CPs at the end of static and dynamic culture and before in vivo implantation. The viability assay employed Calcein fluorescent staining of the grafts. Data are mean ± SEM, *N* = 5 biological replicates per experimental group.

## References

[B1] Akentjew T. L. Terraza C. Suazo C. Maksimcuka J. Wilkens C. A. Vargas F. (2019). Rapid Fabrication of Reinforced and Cell-Laden Vascular Grafts Structurally Inspired by Human Coronary Arteries. Nat. Commun. 10, 3098. 10.1038/s41467-019-11090-3 31308369 PMC6629634

[B2] Albertario A. Swim M. M. Ahmed E. M. Iacobazzi D. Yeong M. Madeddu P. (2019). Successful Reconstruction of the Right Ventricular Outflow Tract by Implantation of Thymus Stem Cell Engineered Graft in Growing Swine. JACC: Basic Transl. Sci. 4, 364–384. 10.1016/j.jacbts.2019.02.001 31312760 PMC6609916

[B3] Alvino V. V. Kilcooley M. Thomas A. C. Carrabba M. Fagnano M. Cathery W. (2020). *In Vitro* and *In Vivo* Preclinical Testing of Pericyte-Engineered Grafts for the Correction of Congenital Heart Defects. J. Am. Heart Assoc. 9, e014214. 10.1161/JAHA.119.014214 32067581 PMC7070228

[B4] Avolio E. Caputo M. Madeddu P. (2015). Stem Cell Therapy and Tissue Engineering for Correction of Congenital Heart Disease. Front. Cel Dev. Biol. 3, 39. 10.3389/fcell.2015.00039 PMC4485350 26176009

[B5] Avolio E. Rodriguez-Arabaolaza I. Spencer H. L. Riu F. Mangialardi G. Slater S. C. (2015). Expansion and Characterization of Neonatal Cardiac Pericytes Provides a Novel Cellular Option for Tissue Engineering in Congenital Heart Disease. J. Am. Heart Assoc. 4, e002043. 10.1161/JAHA.115.002043 26080813 PMC4599542

[B6] Barberà J. A. Peinado V. I. Santos S. Ramirez J. Roca J. Rodriguez-Roisin R. (2001). Reduced Expression of Endothelial Nitric Oxide Synthase in Pulmonary Arteries of Smokers. Am. J. Respir. Crit. Care Med. 164, 709–713. 10.1164/ajrccm.164.4.2101023 11520741

[B7] Bongso A. Fong C.-Y. Gauthaman K. (2008). Taking Stem Cells to the Clinic: Major Challenges. J. Cel. Biochem. 105, 1352–1360. 10.1002/jcb.21957 18980213

[B8] Carrabba M. Madeddu P. (2018). Current Strategies for the Manufacture of Small Size Tissue Engineering Vascular Grafts. Front. Bioeng. Biotechnol. 6, 41. 10.3389/fbioe.2018.00041 29721495 PMC5916236

[B9] Chang W. G. Niklason L. E. (2017). A Short Discourse on Vascular Tissue Engineering. NPJ Regen. Med. 2, 7. 10.1038/s41536-017-0011-6 29057097 PMC5649630

[B10] Cheema F. H. Polvani G. Argenziano M. Pesce M. (2012). Combining Stem Cells and Tissue Engineering in Cardiovascular Repair - a Step Forward to Derivation of Novel Implants with Enhanced Function and Self-Renewal Characteristics. Recent Pat. Cardiovasc. Drug Discov. 7, 10–20. 10.2174/157489012799362403 22280334

[B11] De Visscher G. Mesure L. Meuris B. Ivanova A. Flameng W. (2012). Improved Endothelialization and Reduced Thrombosis by Coating a Synthetic Vascular Graft with Fibronectin and Stem Cell Homing Factor SDF-1α. Acta Biomater. 8, 1330–1338. 10.1016/j.actbio.2011.09.016 21964214

[B12] Durko A. P. Yacoub M. H. Kluin J. (2020). Tissue Engineered Materials in Cardiovascular Surgery: The Surgeon's Perspective. Front. Cardiovasc. Med. 7, 55. 10.3389/fcvm.2020.00055 32351975 PMC7174659

[B13] Ferreiro C. R. Chagas A. C. P. Carvalho M. H. C. Dantas A. P. Jatene M. B. Bento De Souza L. C. (2001). Influence of Hypoxia on Nitric Oxide Synthase Activity and Gene Expression in Children with Congenital Heart Disease. Circulation 103, 2272–2276. 10.1161/01.cir.103.18.2272 11342476

[B14] Gao Y. Chen T. Raj J. U. (2016). Endothelial and Smooth Muscle Cell Interactions in the Pathobiology of Pulmonary Hypertension. Am. J. Respir. Cel Mol Biol. 54, 451–460. 10.1165/rcmb.2015-0323tr PMC4821060 26744837

[B15] Ghorbel M. T. Jia H. Swim M. M. Iacobazzi D. Albertario A. Zebele C. (2019). Reconstruction of the Pulmonary Artery by a Novel Biodegradable Conduit Engineered with Perinatal Stem Cell-Derived Vascular Smooth Muscle Cells Enables Physiological Vascular Growth in a Large Animal Model of Congenital Heart Disease. Biomaterials 217, 119284. 10.1016/j.biomaterials.2019.119284 31255979 PMC6658806

[B16] Gong W. Lei D. Li S. Huang P. Qi Q. Sun Y. (2016). Hybrid Small-Diameter Vascular Grafts: Anti-expansion Effect of Electrospun Poly ε-caprolactone on Heparin-Coated Decellularized Matrices. Biomaterials 76, 359–370. 10.1016/j.biomaterials.2015.10.066 26561933

[B17] Gupta S. (2011). Intention-to-treat Concept: A Review. Perspect. Clin. Res. 2, 109–112. 10.4103/2229-3485.83221 21897887 PMC3159210

[B18] Han J.-A. Seo E. Y. Kim H. J. Park S. J. Yoo H. Y. Kim J. Y. (2013). Hypoxia-augmented Constriction of Deep Femoral Artery Mediated by Inhibition of eNOS in Smooth Muscle. Am. J. Physiol. Cell Physiol. 304, C78–C88. 10.1152/ajpcell.00176.2012 23099643

[B19] Hunter K. S. Albietz J. A. Lee P. F. Lanning C. J. Lammers S. R. Hofmeister S. H. (1985). *In Vivo* measurement of Proximal Pulmonary Artery Elastic Modulus in the Neonatal Calf Model of Pulmonary Hypertension: Development and *Ex Vivo* Validation. J. Appl. Physiol. 108, 968–975. 10.1152/japplphysiol.01173.2009 PMC2853208 20093662

[B20] Hunter K. S. Lee P.-F. Lanning C. J. Ivy D. D. Kirby K. S. Claussen L. R. (2008). Pulmonary Vascular Input Impedance Is a Combined Measure of Pulmonary Vascular Resistance and Stiffness and Predicts Clinical Outcomes Better Than Pulmonary Vascular Resistance Alone in Pediatric Patients with Pulmonary Hypertension. Am. Heart J. 155, 166–174. 10.1016/j.ahj.2007.08.014 18082509 PMC3139982

[B21] Iacobazzi D. Swim M. M. Albertario A. Caputo M. Ghorbel M. T. (2018). Thymus-Derived Mesenchymal Stem Cells for Tissue Engineering Clinical-Grade Cardiovascular Grafts. Tissue Eng. Part A 24, 794–808. 10.1089/ten.tea.2017.0290 29054134

[B22] Iacobazzi D. Rapetto F. Albertario A. Swim M. M. Narayan S. Skeffington K. (2021). Wharton's Jelly-Mesenchymal Stem Cell-Engineered Conduit for Pediatric Translation in Heart Defect. Tissue Eng. A 27, 201–213. 10.1089/ten.tea.2020.0088 32571164

[B23] Jover E. Fagnano M. Cathery W. Slater S. Pisanu E. Gu Y. (2021). Human Adventitial Pericytes Provide a Unique Source of Anti-calcific Cells for Cardiac Valve Engineering: Role of microRNA-132-3p. Free Radic. Biol. Med. 165, 137–151. 10.1016/j.freeradbiomed.2021.01.029 33497799

[B24] Kim H. J. Jang J. H. Zhang Y. H. Yoo H. Y. Kim S. J. (2019). Fast Relaxation and Desensitization of Angiotensin II Contraction in the Pulmonary Artery via AT1R and Akt-Mediated Phosphorylation of Muscular eNOS. Pflugers Arch. Eur. J. Physiol. 471, 1317–1330. 10.1007/s00424-019-02305-z 31468138

[B25] Lee K.-W. Johnson N. R. Gao J. Wang Y. (2013). Human Progenitor Cell Recruitment via SDF-1α Coacervate-Laden PGS Vascular Grafts. Biomaterials 34, 9877–9885. 10.1016/j.biomaterials.2013.08.082 24060423 PMC3882008

[B26] Lenoir M. Pontailler M. Gaudin R. Gerelli S. Tamisier D. Bonnet D. (2017). Outcomes of Palliative Right Ventricle to Pulmonary Artery Connection for Pulmonary Atresia with Ventricular Septal Defect†. Eur. J. Cardiothorac. Surg. 52, 590–598. 10.1093/ejcts/ezx194 28633393

[B27] Luo J. Qin L. Zhao L. Gui L. Ellis M. W. Huang Y. (2020). Tissue-Engineered Vascular Grafts with Advanced Mechanical Strength from Human iPSCs. Cell Stem Cell 26, 251–261. 10.1016/j.stem.2019.12.012 31956039 PMC7021512

[B28] Ma X. He Z. Li L. Liu G. Li Q. Yang D. (2017). Development and *In Vivo* Validation of Tissue-Engineered, Small-Diameter Vascular Grafts from Decellularized Aortae of Fetal Pigs and Canine Vascular Endothelial Cells. J. Cardiothorac. Surg. 12, 101. 10.1186/s13019-017-0661-x 29178903 PMC5702065

[B29] Mahapatra S. Nishimura R. A. Sorajja P. Cha S. McGoon M. D. (2006). Relationship of Pulmonary Arterial Capacitance and Mortality in Idiopathic Pulmonary Arterial Hypertension. J. Am. Coll. Cardiol. 47, 799–803. 10.1016/j.jacc.2005.09.054 16487848

[B30] Majid Q. A. Fricker A. T. R. Gregory D. A. Davidenko N. Hernandez Cruz O. Jabbour R. J. (2020). Natural Biomaterials for Cardiac Tissue Engineering: A Highly Biocompatible Solution. Front. Cardiovasc. Med. 7, 554597. 10.3389/fcvm.2020.554597 33195451 PMC7644890

[B31] Manavitehrani I. Ebrahimi P. Yang I. Daly S. Schindeler A. Saxena A. (2019). Current Challenges and Emergent Technologies for Manufacturing Artificial Right Ventricle to Pulmonary Artery (RV-PA) Cardiac Conduits. Cardiovasc. Eng. Tech. 10, 205–215. 10.1007/s13239-019-00406-5 30767113

[B32] McCoy E. (2017). Understanding the Intention-To-Treat Principle in Randomized Controlled Trials. WestJEM 18, 1075–1078. 10.5811/westjem.2017.8.35985 29085540 PMC5654877

[B33] Mewhort H. E. M. Turnbull J. D. Satriano A. Chow K. Flewitt J. A. Andrei A.-C. (2016). Epicardial Infarct Repair with Bioinductive Extracellular Matrix Promotes Vasculogenesis and Myocardial Recovery. J. Heart Lung Transplant. 35, 661–670. 10.1016/j.healun.2016.01.012 26987597

[B34] Mewhort H. E. M. Svystonyuk D. A. Turnbull J. D. Teng G. Belke D. D. Guzzardi D. G. (2017). Bioactive Extracellular Matrix Scaffold Promotes Adaptive Cardiac Remodeling and Repair. JACC: Basic Transl. Sci. 2, 450–464. 10.1016/j.jacbts.2017.05.005 30062163 PMC6034485

[B35] Pappa K. I. Anagnou N. P. (2009). Novel Sources of Fetal Stem Cells: where Do They Fit on the Developmental Continuum? Regenerative Med. 4, 423–433. 10.2217/rme.09.12 19438317

[B36] Pashneh-Tala S. MacNeil S. Claeyssens F. (2016). The Tissue-Engineered Vascular Graft-Past, Present, and Future. Tissue Eng. B Rev. 22, 68–100. 10.1089/ten.teb.2015.0100 PMC4753638 26447530

[B37] Percie du Sert N. Hurst V. Ahluwalia A. Alam S. Avey M. T. Baker M. (2020). The ARRIVE Guidelines 2.0: Updated Guidelines for Reporting Animal Research. Plos Biol. 18, e3000410. 10.1371/journal.pbio.3000410 32663219 PMC7360023

[B38] Ran X. Ye Z. Fu M. Wang Q. Wu H. Lin S. (2019). Design, Preparation, and Performance of a Novel Bilayer Tissue-Engineered Small-Diameter Vascular Graft. Macromol Biosci. 19, e1800189. 10.1002/mabi.201800189 30259649

[B39] Smith R. J. Jr. Nasiri B. Kann J. Yergeau D. Bard J. E. Swartz D. D. (2020). Endothelialization of Arterial Vascular Grafts by Circulating Monocytes. Nat. Commun. 11, 1622. 10.1038/s41467-020-15361-2 32238801 PMC7113268

[B40] Swim M. M. Albertario A. Iacobazzi D. Caputo M. Ghorbel M. T. (2019). Amnion-Based Scaffold with Enhanced Strength and Biocompatibility for *In Vivo* Vascular Repair. Tissue Eng. Part A 25, 603–619. 10.1089/ten.tea.2018.0175 30284966

[B41] Syedain Z. Reimer J. Lahti M. Berry J. Johnson S. Bianco R. (2016). Tissue Engineering of Acellular Vascular Grafts Capable of Somatic Growth in Young Lambs. Nat. Commun. 7, 12951. 10.1038/ncomms12951 27676438 PMC5052664

[B42] Syedain Z. Haynie B. Johnson S. Lahti M. Berry J. Carney J. (2021). Tissue Remodeling of Engineered Valved Conduit Evaluated at 52 Weeks in the Growing Lamb. Struct. Heart 5, 13. 10.1080/24748706.2021.1898262

[B43] Yuan Y. Khan S. Stewart D. J. Courtman D. W. (2020). Engineering Blood Outgrowth Endothelial Cells to Optimize Endothelial Nitric Oxide Synthase and Extracellular Matrix Production for Coating of Blood Contacting Surfaces. Acta Biomater. 109, 109–120. 10.1016/j.actbio.2020.04.016 32302726

