# Supplementary figures and images for: Reconstruction of the Swine Pulmonary Artery Using a Graft Engineered With Syngeneic Cardiac Pericytes
Source: Front Bioeng Biotechnol. 2021 Sep 9;9:715717. doi: 10.3389/fbioe.2021.715717 (PMC8459923; doi:10.3389/fbioe.2021.715717)

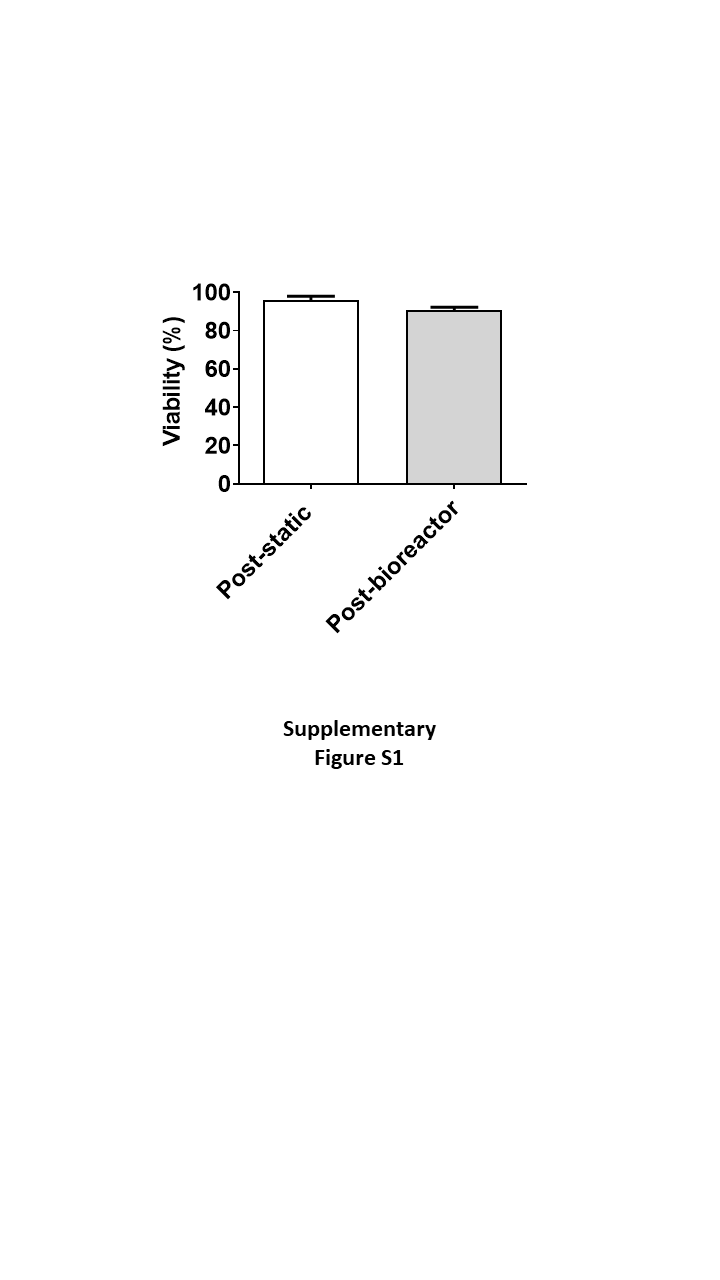

Supplement: Supplementary file 1 [file Image1.TIF]
